# Aluminum-doped gallium sulfide shell for enhancing the luminescence properties of Ag-In-Ga-S core quantum dots and their composite for dye adsorption

**DOI:** 10.1039/d6ra00895j

**Published:** 2026-07-02

**Authors:** Manunya Tepakidareekul, Supinya Nijpanich, Vasuphat Tunsound, Phitsamai Kamonpha, Netsirin Gissawong, Aissara Rasritat, Worawat Meevasana

**Affiliations:** a School of Physics, Institute of Science, Suranaree University of Technology Nakhon Ratchasima 30000 Thailand; b Synchrotron Light Research Institute (Public Organization) Nakhon Ratchasima 30000 Thailand; c National Institute of Reference Laboratories, Department of Science Service Bangkok 10400 Thailand

## Abstract

High-performance, environmentally friendly AgIn_*x*_Ga_1−*x*_S_2_/GaS_*y*_ core/shell quantum dots (QDs) are needed for applications including solar cells and photocatalysis. These QDs offer greater durability than organic dyes and strong UV-visible absorption, enabling tuning of the titanium dioxide (TiO_2_) bandgap by adjusting the particle size or chemical composition to harvest more of the solar spectrum. However, the inherently amorphous GaS_*y*_ shell is vulnerable to environmental damage, thus limiting stability. Here, a simple aluminum (Al)-doping strategy for the GaS_*y*_ shell is demonstrated, where the incorporation of Al^3+^ ions into the GaS_*y*_ shell increases its thickness and the subsequent partial oxidation, yielding a protective aluminum oxide (Al_2_O_3_) layer. The formation of Al_2_O_3_ enhances photoluminescence quantum yield and long-term stability by shielding the QDs from degradation and reducing defect states within the GaS_*y*_ shell. Additionally, the self-oxidized Al_2_O_3_ would modify the local electronic structure of the GaS_*y*_ shell, resulting in a red shift in emission. When AgIn_*x*_Ga_1−*x*_S_2_/GaS_*y*_ : Al core/shell QDs were fabricated with P25–TiO_2_, the QDs/TiO_2_ composite was evaluated for the adsorption of methyl orange as a model pollutant. The adsorption isotherms fit the Freundlich model, indicating a heterogeneous surface due to QD deposition on P25–TiO_2_. The composite displayed substantially higher removal efficiency than bare P25–TiO_2_. Kinetic analysis results followed a pseudo-second-order model, consistent with a chemisorption mechanism between the composite and dye. Therefore, the composite exhibits excellent dye adsorption performance, laying a solid foundation for the subsequent development of photocatalytic systems.

## Abstract 2

Mechanism of photoluminescence properties enhancement in AgIn*_x_*Ga_1-*x*_S_2_/GaS_*y*_ core/shell quantum dots (QDs) via aluminum doping.

## Graphical abstract

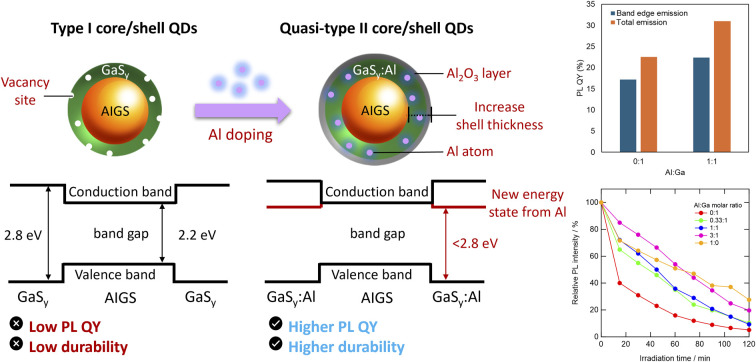

## Introduction

Synthetic organic dyes, such as methyl orange, methylene blue, and rhodamine B, are extensively used in several industries.^1–3^ The contamination of wastewater by these dyes poses a significant threat to aquatic environments and, consequently, to humans and animals through consumption. This is due to the dyes' nonbiodegradable nature, toxicity, chemical stability, mutagenicity, and carcinogenicity.^4^ Therefore, the removal of these dyes from wastewater is a matter of high practical significance. Currently, the adsorption-photocatalysis process is very attractive due to its cost-effectiveness, minimal secondary waste production, and environmental friendliness.^5,6^

Titanium dioxide (TiO_2_) is a well-known photocatalyst, which is popularly used for wastewater treatment owing to its high stability, low cost, good biocompatibility, and environmental friendliness.^7,8^ However, its application is limited to the ultraviolet light region due to its broad bandgap energy and high recombination rate of photoexcited charges.^9,10^ To address the limitations of bare TiO_2_, researchers are increasingly focusing on preparing the composite between TiO_2_ and other materials to integrate TiO_2_/semiconductor quantum dot (QD) composites, leveraging the tunable bandgap of the QDs to enhance performance. The integration of tunable-bandgap QDs with TiO_2_ is a burgeoning strategy aimed at bypassing the inherent constraints of pristine TiO_2_.^11,12^ Furthermore, the fabrication of heterojunctions/composites is an efficient method to integrate several functionalities and achieve novel properties in TiO_2_-based system.^13^ Prior to the photocatalytic degradation of organic contaminants, the target molecules must first reach adsorption–desorption equilibrium on the photocatalyst surface. Therefore, the adsorption process plays a crucial role in the overall reaction.

QDs are semiconductor nanocrystals that have discrete electronic energy levels like isolated atoms, resulting in differences in chemical and physical properties compared to the bulk material.^14,15^ The quantum confinement effect plays a crucial role in determining their band gap energies; thereby, the optical properties of QDs can be easily tuned by controlling their size as well as their chemical composition.^16,17^ Due to the bandgap tunability of these materials, a variety of potential applications of QDs have been proposed, including display, chemosensors, bioimaging, and photocatalysts.^18–26^ During the past few decades, cadmium-containing QDs such as cadmium sulfide (CdS), cadmium selenide (CdSe), and cadmium telluride (CdTe) have been intensively studied. However, the applications of these QDs in practical devices have been limited because of the toxicity of cadmium. In addition to inherent toxicity, these QDs also suffer significantly from self-absorption, which also restrains them from many applications.^17,27^ Silver indium sulfide (AgInS_2_) QDs, which are cadmium-free QDs, have been considered emerging alternatives to toxic conventional QDs due to their lower toxicity. Moreover, they exhibit a large Stokes shift with a longer photoluminescence (PL) lifetime. However, these QDs exhibit a broad PL spectrum due to defects in the crystal and/or on the surface of QDs, resulting in a deficiency in monochromaticity.^28–30^ Zinc sulfide (ZnS), a large-bandgap material, has been utilized to cover the surface of AgInS_2_. However, rather than generating type-I core/shell QDs, the Zn^2+^ ions alter the surface defect levels through surface passivation with ZnS and/or the partial alloying resulting from cation exchange from In^3+^ to Zn^2+^, resulting in a drastic blue shift in PL.^31^ Consequently, the new shell material necessitates investigation to address the issue. In 2018, Uematsu *et al.* reported that coating the AgInS_2_ core QDs surface with gallium sulfide (GaS_*y*_) can reduce surface defects of the QDs, leading to the spectrally narrow band-edge emission in the yellow region (580 nm) with a moderately high PL quantum yield (QY).^32^ This coating process has also been extensively applied in quaternary silver indium gallium sulfide (AgIn_*x*_G_1−*x*_S_2_) core QDs, which possess a larger bandgap compared to AgInS_2_ core QDs. These QDs exhibit green emission with narrow band emission after GaS_*y*_ coating.^33^ Although the GaS_*y*_ coating can overcome the issue of PL properties, the structure of GaS_*y*_ is still a problem due to its amorphous structure. Amorphous materials typically exist in a higher energy state than crystalline materials. Therefore, core/shell QDs with amorphous shells are expected to be less stable than those protected by crystalline shells. In fact, exposure to light, moisture, and oxygen readily damages the AgIn_*x*_G_1−*x*_S_2_/GaS_*y*_ core/shell QDs, which lowers their PL performance. Therefore, it is necessary to find a simple and efficient method to improve the stability of these nanoparticles. Typically, there are several approaches that enhance the PL properties and stability by utilizing surface modifications. For example, the addition of stabilizing agents that preferentially bind to the defect sites of the shell, including metal ions or passivation ligands, can improve PL properties.^34,35^ Coating the core/shell QDs with polymer^36,37^ or metal–organic frameworks (MOFs)^38–40^ is also an effective strategy for protecting QDs from the environment. In addition, doping self-passivating metals into the shell structure is a promising approach that can protect the exposed metal surface from oxidation and other undesirable chemical reactions. In 2013, Li *et al.* introduced a facile method to enhance the photostability of CdSe/CdS core/shell QDs by doping aluminum (Al) into the CdS shell. The results showed that Al^3+^ ions in the coating shell were oxidized to form an aluminum oxide (Al_2_O_3_) layer that can serve as a self-passivation layer on the surface of the core/shell QDs and effectively prevented further photodegradation during long-term light irradiation.^41^ This method is also effective in CuInS_2_/ZnS core/shell QDs. The results show a similar Al_2_O_3_ passivation layer that was formed on the surface of ZnS, which can protect the core/shell QDs and enhance PL.^42^ Consequently, aluminum is a highly promising material that easily undergoes self-oxidation to form a protective layer, which is essential for the practical application of QDs. Furthermore, the substantial band gap of this material (7.0–7.6 eV)^43^ may serve as a barrier that diminishes non-radiative recombination.^44^ In addition, the Al doping may increase the photocatalytic efficacy for dye degradation, which is crucial for subsequent applications.^45^ Given these benefits, the development of the GaS_*y*_ shell through Al doping is anticipated.

To the best of our knowledge, this work represents the first example of Al doping into the GaS_*y*_ shell. Al doping increased the shell thickness of the QDs, and the doped Al^3+^ ions underwent self-oxidation, leading to Al_2_O_3_ formation, which enhanced the durability and PL properties of AgIn_*x*_G_1−*x*_S_2_/GaS_*y*_ core/shell QDs. In addition, the QDs/TiO_2_ composite was prepared to investigate the impact of the AgIn_*x*_G_1−*x*_S_2_/GaS_*y*_ : Al core/shell QDs on TiO_2_, concerning the dye adsorption mechanism between the composite and methyl orange. This experiment lays the groundwork for further adsorption-photocatalytic applications of these QDs.

## Experimental section

### Materials

Silver acetate (AgOAc) was purchased from Mitsuwa Chemicals Co., Ltd. Gallium(iii) acetylacetonate (Ga[acac]_3_) and indium(iii) acetate (In[OAc]_3_) were purchased from Sigma-Aldrich. Gallium(iii) chloride (GaCl_3_), diethylammonium diethyldithiocarbamate (DEA-DDTC), and aluminum isopropoxide (Al[*i*-PrO]_3_) were supplied by Fujifilm Wako Chemicals. Dimethylthiourea (DMTU) was purchased from TCI. Aeroxide®. Titanium dioxide P25 (P25–TiO_2_) was purchased from Evonik. Methyl orange was purchased from LOBA CHEMIE PVT. LTD. Hydrochloric acid (37% w/w) was purchased from Kishida Chemical Co., Ltd. Acetone, chloroform, methanol, ethanol, and hexane were obtained from Merck. Oleylamine (OLA) was purchased from Aldrich and used after vacuum distillation in the presence of calcium hydride. Water used in the present study was purified using a Milli-Q Integral 3 (Merck) with a resistivity of greater than 18.2 MΩ cm.

### Instruments

The reaction mixture temperature was directly measured using a thermocouple and controlled using a heating mantle equipped with a proportional–integral–derivative controller. The ultraviolet-visible (UV-vis) absorption and PL spectra of the samples were recorded using a double-beam UV-vis spectrophotometer (JASCO, Japan, V-670) and a fluorescence spectrometer (JASCO, Japan, FP-8600), respectively. The PL QYs were measured using a diode-array spectrometer equipped with an integrating sphere (Hamamatsu, Japan, PMA12). The fluorescence decay processes were recorded using a time-correlated single-photon counting setup (Hamamatsu, Japan, Quantaurus-Tau). The size and morphology of QDs were observed using a TEM instrument (Hitachi, Japan, H-7650) at an acceleration voltage of 100 kV. Carbon-coated copper TEM grids (Oken-shoji, Japan, HRC-C10) were used to prepare the TEM samples. XRD patterns were obtained using a powder X-ray diffractometer (Rigaku, Japan, SmartLab) equipped with a parallel beam/parallel slit analyser. XPS spectra were recorded using an X-ray photoelectron spectrometer (Shimadzu, KRATOS AXIS-165x) equipped with a monochromatized Al-Kα X-ray source (*h*ν = 1486.6 eV). The C 1s spectrum was used as the peak reference, marking the C–C/C–H at 284.4 eV. The chemical compositions of the precursors and QDs were obtained using energy-dispersive X-ray analysis (AMETEK EDAX X1).

### Synthetic procedure

Synthesis of gallium diethyldithiocarbamate (Ga[DDTC]_3_) precursor. Ga(DDTC)_3_ was synthesized according to the literature, with a slight modification.^46^ Typically, GaCl_3_ (5.6 mmol) and DEA-DDTC (17.1 mmol) were mixed with toluene (75 mL). The mixture solution was stirred under a N_2_ atmosphere at room temperature for 3 h. White crystals were collected, recrystallized and dried *in vacuo* at 50 °C for 2 h and then characterized by EDX.

#### Synthesis of AgInxG1_−_xS2 core QDs

The OLA-capped AgIn_*x*_G_1−*x*_S_2_ core QDs were synthesized according to the literature, with a slight modification.^47^ Typically, Ga(DDTC)_3_ (0.4 mmol) and In(OAc)_3_ (0.15 mmol) were mixed with OLA (10 mL) in a two-necked round-bottom flask. The mixture was degassed under vacuum at 80 °C for 5 min. Under an argon atmosphere, the solution was heated to 130 °C at a rate of 50 °C min^−1^, followed by the injection of 1 mL of Ag(OAc) (0.2 M) dissolved in OLA. The solution temperature was increased to 180 °C and kept at this temperature for 20 min. The solution was then cooled to room temperature and 6 mL of acetone was added to remove excess Ga(DDTC)_3_. For the purification process, the AgIn_*x*_G_1−*x*_S_2_ core QDs were precipitated by the smallest possible amount of methanol, followed by centrifugation, and the precipitate was redispersed in hexane.

#### Synthesis of AgIn_*x*_G_1−*x*_S_2_/GaS_*y*_ and AgIn_*x*_G_1−*x*_S_2_/GaS_*y*_

Al core/shell QDs with various Al/Ga molar ratios. The OLA-capped AgIn_*x*_G_1−*x*_S_2_/GaS_*y*_ core/shell QDs were synthesized according to the literature, with a slight modification.^32^ In a two-necked round-bottom flask, 30 nmol of AgIn_*x*_G_1−*x*_S_2_ core QDs, Ga(acac)_3_ (0.1 mmol) and DMTU (0.1 mmol) were mixed in OLA (10 mL). The mixture was degassed under vacuum at 75 °C for 5 min. Under an argon atmosphere, the solution was heated to 230 °C at a rate of approximately 150 °C min^−1^, and then the temperature was increased to 280 °C at a rate of 2 °C min^−1^. The solution color changed from orange to yellow, indicating GaS_*y*_ formation at the core QD surface. The solution was cooled to room temperature, followed by the addition of HCl (50 µL, 37%). The solution was degassed under vacuum for 5 min and rapidly heated to 260 °C at a rate of approximately 150 °C min^−1^. The temperature was maintained for 30 min and then cooled to room temperature. The solution was centrifuged to remove large particles and purified using the above-mentioned process. The synthesis of the AgIn_*x*_G_1−*x*_S_2_/GaS_*y*_ : Al core/shell QDs was basically similar to that of AgIn_*x*_G_1−*x*_S_2_/GaS_*y*_ QDs. Various amounts of Al(*i*-PrO)_3_ were mixed with Ga(acac)_3_ and DMTU in OLA; the amount of Al(*i*-PrO)_3_ varied from 0.025 to 0.05, 0.075, and 0.1 mmol, corresponding to 1 : 3, 1 : 1, 3 : 1, and 1 : 0 Al/Ga precursor molar ratios, respectively.

#### Synthesis of AgIn_*x*_G_1−*x*_S_2_/GaS_*y*_

Al core/shell QDs with various amounts of shell precursors. The synthesis of the AgIn_*x*_G_1−*x*_S_2_/GaS_*y*_ : Al core/shell QDs with different amounts of shell precursor was similar to that mentioned above. The 1 : 1 molar ratio of Al and Ga was selected for synthesis due to the highest PL QY, while the total amount of Ga(acac)_3_ and Al(*i*-PrO)_3_ was varied from 0.1 to 0.3 mmol, as well as the amount of DMTU.

#### Synthesis of AgIn_*x*_G_1−*x*_S_2_/GaS_*y*_

Al core/shell QDs and P25–TiO_2_ (QDs@P25–TiO_2_) composites. First, the QD solution was purified by precipitation and centrifugation to remove OLA from the surface of QDs. Then, the obtained QD powder was dried at 60 °C overnight. After that, a mixture of 0.2 g P25–TiO_2_ powder and 2 mL ethanol was mixed and ground with various quantities of purified QD powder (20, 40, and 60 mg) for 30 min until the mixture was dried to powder. The powder mixture was then dried at 60 °C for 3 h.

### Adsorption test

The standard solutions of methyl orange were diluted with deionized water to various concentration gradients. The concentration gradients of the standard curve for methyl orange were 0, 2, 4, 6, 8, and 10 mg L^−1^. The absorbance of the solutions was measured at 465 nm, which is the maximum absorption wavelength for this dye. Based on the measurements, a standard curve equation was established. The adsorption of methyl orange on the QDs@P25–TiO_2_ composites was conducted in aqueous solution under dark conditions. Typically, 25 mg of QDs@P25–TiO_2_ was added to 100 mL of 10 mg L^−1^ methyl orange solution under vigorous stirring (600 rpm) for a certain time. Following a specified interval of adsorption, aliquots were collected and centrifuged at a speed of 9000 rpm for 3 min. The supernatant was collected and analyzed using a UV-vis spectrophotometer. The adsorption capacity (*q*), at time (*t*), the saturated adsorption capacity (*q*_e_), and the removal rate (*R*) of methyl orange for the QDs@P25–TiO_2_ adsorbent were calculated according to the following equations:1
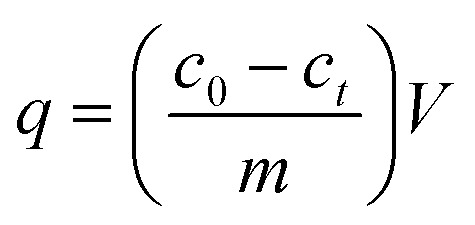
2
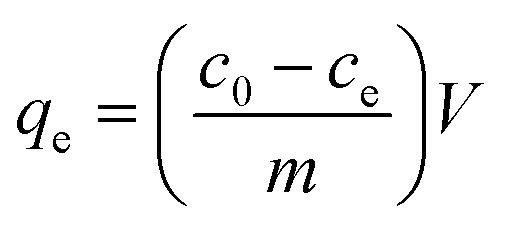
3
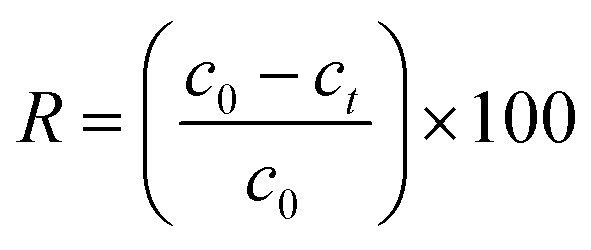
where *c*_0_ is the initial mass concentration of methyl orange before adsorption (g L^−1^); *c*_*t*_ is the mass concentration of methyl orange at time *t* during adsorption (g L^−1^); *c*_e_ is the equilibrium concentration of methyl orange after adsorption (g L^−1^); *V* is the volume of the dye solution to be adsorbed (L); and *m* is the amount of adsorbent used (mg).

The initial concentrations of methyl orange were varied from 5 to 40 mg L^−1^ to further investigate the adsorption behavior and kinetics of QDs@P25–TiO_2_ towards the dye. At the end of the adsorption time (80 min), the residual concentrations of methyl orange were measured, and the adsorption capacity, *q*_e_, was determined. The results were fitted to the Langmuir and the Freundlich isotherm models^48^ to investigate and describe the adsorption behavior of the QDs@P25–TiO_2_ composite toward methyl orange, as shown in the following equations:

Langmuir adsorption isotherm model:4
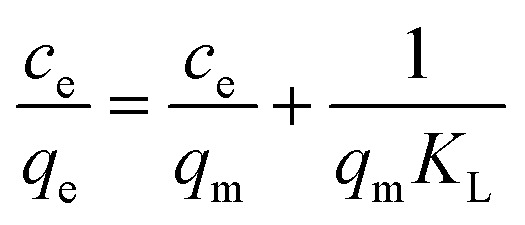


Freundlich adsorption isotherm model:5
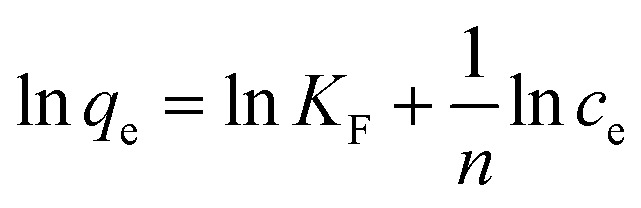
where *q*_e_ is the equilibrium adsorption capacity of the adsorbent for the dye (mg L^−1^); *q*_m_ is the maximum adsorption capacity of the adsorbent (mg L^−1^); *K*_L_ is the Langmuir adsorption constant (L mg^−1^); *K*_F_ is the Freundlich adsorption constant (L mg^−1^); and 1/*n* is the adsorption intensity, and a larger value of *n* indicates a more favorable adsorption process.

In the case of a kinetic study in the process of QDs@P25–TiO_2_ adsorbing methyl orange, pseudo-first-order and pseudo-second-order kinetic models were employed to analyze and describe the kinetic process of dye adsorption.^49^

Pseudo-first order kinetic model:

6 ln(*q*_e_ − *q*_t_) = ln *q*_e_ − *k*_1_*t*

Pseudo-second order kinetic model:7
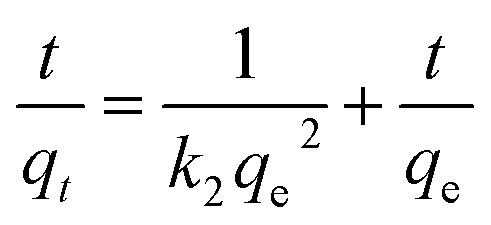


## Results and discussion

### Effects of different Ga/Al molar ratios for the shell coating on the optical properties and morphology

**Fig. 1:**
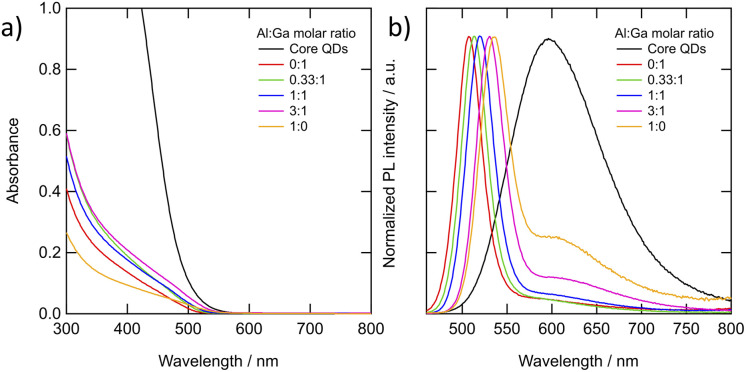
(a) UV-vis absorption and (b) intensity-normalized PL spectra for AgIn_*x*_Ga_1−*x*_S_2_, AgIn_*x*_Ga_1−*x*_S_2_/GaS_*y*_, and AgIn_*x*_Ga_1−*x*_S_2_/GaS_*y*_ : Al QDs synthesized with varying Al : Ga molar ratios. Black line, core QDs; red line, 0 : 1; green line, 0.33 : 1; blue line, 1 : 1; pink line, 3 : 1; and yellow line, 1 : 0.

**Fig. 2:**
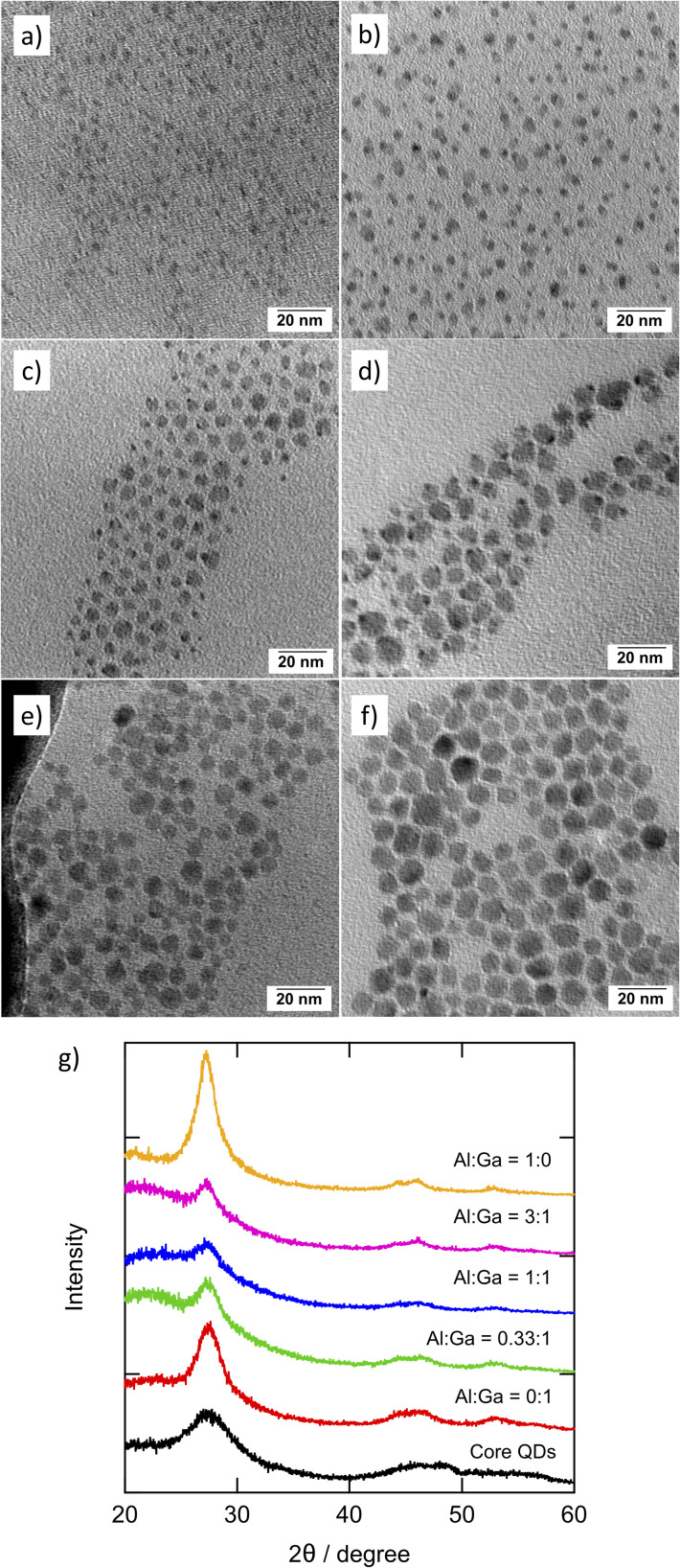
TEM images of (a) AgIn_*x*_Ga_1−*x*_S_2_ core QDs and AgIn_*x*_Ga_1−*x*_S_2_/GaS_*y*_ : Al core/shell QDs with (b) 1 : 0, (c) 0.33 : 1, (d) 1 : 1, (e) 3 : 1, and (f) 0 : 1 Al/Ga molar ratios, and (g) their XRD patterns.

The influence of stoichiometry on the optical properties and morphologies of AgIn_*x*_G_1−*x*_S_2_/GaS_*y*_ : Al core/shell QDs was investigated using the samples synthesized with different Al/Ga precursor molar ratios for the shell coating, namely, 0 : 1, 0.33 : 1, 1 : 1, 3 : 1, and 1 : 0. Fig. 1 depicts the changes in optical properties of the QDs. First, the UV-vis absorption spectra shifted to a shorter wavelength when AgIn_*x*_G_1−*x*_S_2_ core QDs were coated with GaS_*y*_ shells. Simultaneously, the spectrally broad PL of AgIn_*x*_G_1−*x*_S_2_ core QDs totally changed to the narrow band-edge emission centered at 507 nm; the full width at half maxima (FWHM) for the PL spectra decreased from 116 nm to 32 nm. The PL QY of the QDs increased from 13.8–22.5%, and the value when the wavelength region was limited to the band edge emission was 17.2%. Fig. 2a and b depict TEM images of AgIn_*x*_G_1−*x*_S_2_ core QDs and AgIn_*x*_G_1−*x*_S_2_/GaS_*y*_ core/shell QDs, respectively. The QDs exhibited the same spherical shape as the size increased from 3.7 nm to 4.3 nm. These results indicate that the defect sites on the surface of the AgIn_*x*_G_1−*x*_S_2_ core QDs were almost eliminated by coating with GaS_*y*_ shells. However, the PL QYs of AgIn_*x*_G_1−*x*_S_2_/GaS_*y*_ QDs were quite low compared to AgInS_2_/GaS_*y*_ QDs due to the larger energy gap of the alloy AgIn_*x*_G_1−*x*_S_2_ core compared to AgInS_2_ [1.8 eV].^50^ This difference would cause more non-radiative recombination processes after GaS_*y*_ shell coating. Moreover, it would be more difficult to coat the GaS_*y*_ shell on the surface of AgIn_*x*_G_1−*x*_S_2_ core QDs compared to AgInS_2_ core QDs.^32^ Therefore, the composition tuning of the GaS_*y*_ shell by Al doping would prevent the non-radiative recombination process due to Al_2_O_3_ formation, leading to the enhanced optical properties and durability of AgIn_*x*_G_1−*x*_S_2_ core QDs. By varying the Al/Ga molar ratio for the GaS_*y*_ shell coating, the absorption and PL spectra were both continuously red-shifted to a longer wavelength with the increment of Al/Ga ratios, as shown in Fig. 1a and b. Moreover, the shell thicknesses, which were evaluated by the differences between the particle sizes of core QDs and core/shell QDs, increased from 0.6 nm to 3.9 nm, as shown in Fig. 2c–e. According to this evidence, two possible factors involved in the red-shift in optical spectra towards Al doping are the increase in the particle size and the modification of the local electronic structure of the GaS_*y*_ shell. The former can be demonstrated by the increase in the shell thickness, as shown in Table 1. The shell thickness dramatically increased from 0.65 nm to 3.89 when the Al/Ga molar ratio was increased from 0 : 1 to 0.33 : 1. Generally, when the shell thickness is increased, a red-shift in the optical spectra is expected due to a decrease in the quantum size effect. The latter factor may be attributed to the presence of Al atoms, which introduce a new energy state in the band alignment of shell materials.^51,52^ This phenomenon may transform the character of type I band alignment, where both the lowest energy electron and hole wavefunctions are confined in the core to quasi-type II core/shell QDs, where holes are well confined in the AgIn_*x*_G_1−*x*_S_2_ core and electrons are delocalized among the AgIn_*x*_G_1−*x*_S_2_ core and GaS_*y*_ : Al.^53,54^

**Table 1:** Relevant parameters of AgIn_*x*_Ga_1−*x*_S_2_ core QDs, AgIn_*x*_Ga_1−*x*_S_2_/GaS_*y*_, and AgIn_*x*_Ga_1−*x*_S_2_/GaS_*y*_ : Al core/shell QDs synthesized with various Al/Ga molar ratios. (The emission ranges of band edge emission = 450–570 nm and total emission = 450–800 nm)

Al/Ga molar ratio	Peak location (nm)	FWHM (nm)	PLQY (%)		Particle size (nm)	Shell thickness (nm)	*E*_g_ (eV)
			Band edge	Total			
Core NPs	598	116	—	13.8	3.7 ± 0.5	—	—
0 : 1	507	32	17.2	22.5	4.3 ± 1.0	0.0.6	2.32
0.33 : 1	512	34	20.9	27.4	5.3 ± 1.2	1.6	2.28
1 : 1	520	35	22.4	31.0	5.9 ± 1.5	2.2	2.23
3 : 1	531	36	15.5	24.8	7.6 ± 1.9	3.9	2.17
1 : 0	536	35	10.1	16.8	9.2 ± 1.7	—	2.12

EDX analyses (Table 2) show that the atomic ratios of Al were 0, 0.27, 0.46, and 0.67, corresponding to 1 : 0, 3 : 1, 1 : 1, and 0.33 : 1 for the Ga/Al molar ratio, respectively, which is quite similar to the nominal Al atomic ratio (0, 0.25, 0.5, and 0.75) but different from the CsPbBr/ZnAlS^55^ and CuInS_2_/ZnAlS core/shell QDs.^56^ In the case of Al doping into the ZnS shell, the quantity of Al^3+^ ions, which can be incorporated with Zn^2+^ ions to form a ZnAlS alloy shell, was very small due to the large difference in ionic radius between Al^3+^ (68 pm) and Zn^2+^ (88 pm). As a result, there was no change in particle size when Al was doped into the ZnS shell. On the contrary, because Al^3+^ and Ga^3+^ (76 pm) have comparable ionic radii, they can readily create an alloy shell that increases particle size. Moreover, it was shown that despite the reduction in the Ga(acac)_3_ ratio, the Ga content remained constant. This finding suggested that GaS_*y*_ : Al formation also implicated the residual Ga(DDTC)_3_ on the AgIn_*x*_G_1−*x*_S_2_ core surface, leading to a remarkably thicker shell.^47^ However, when the Al/Ga molar ratio was increased to 1 : 0, only a tiny amount of Al was found to be 0.07, even though the particle size and shell thickness were increased to 9.2 ± 1.7 and 5.6 nm, respectively. The residual Ga(DDTC)_3_ on the AgIn_*x*_G_1−*x*_S_2_ core surface may have been insufficient to create a complete GaS_*y*_ : Al alloy shell, resulting in Ostwald ripening of the AgIn_*x*_G_1−*x*_S core during shell synthesis.^57,58^

**Table 2:** Atomic concentrations of AgIn_*x*_Ga_1−*x*_S_2_ core QDs and AgIn_*x*_Ga_1−*x*_S_2_/GaS_*y*_ : Al core/shell QDs synthesized using various Al/Ga molar ratios

Al/Ga molar ratio	Al/Ga (mmol)	Elements				
		Al	S	Ag	In	Ga
Core NPs	—	0.00	4.91	1.00	0.75	2.80
0 : 1	0 : 0.1	0.00	2.55	1.00	0.36	1.83
0.33 : 1	0.025 : 0.075	0.27	2.46	1.00	0.44	1.85
1 : 1	0.05 : 0.05	0.46	2.68	1.00	0.50	1.97
3 : 1	0.075 : 0.025	0.67	2.44	1.00	0.51	1.58
1 : 0	0.1 : 0	0.07	2.48	1.00	0.61	1.48

Fig. 2g illustrates the powder X-ray diffraction patterns of the AgIn_*x*_G_1−*x*_S_2_ core and AgIn_*x*_G_1−*x*_S_2_/GaS_*y*_ : Al core/shell QDs with various Al/Ga molar ratios. All of them exhibited three similar peaks corresponding to the (112), (204), and (312) planes of the AgIn_*x*_G_1−*x*_S_2_ core QDs structure,^33^ whereas the planes of GaS_*y*_ or Al_2_O_3_ were not observed in XRD patterns. These results confirm that Al doping in the amorphous GaS_*y*_ shell did not exchange with core elements or form any Al_2_O_3_ crystals.

**Fig. 3:**
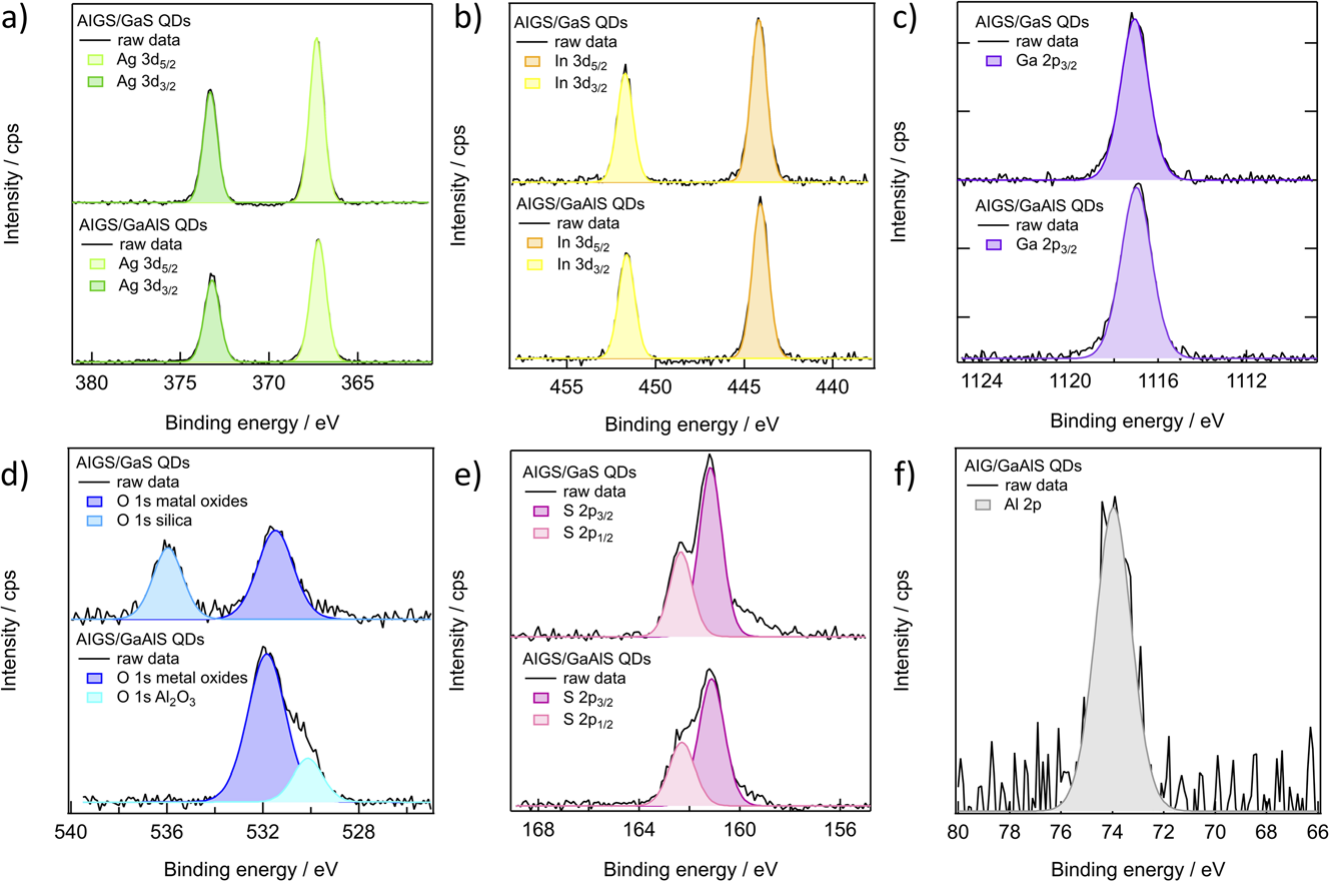
XPS spectra corresponding to (a) Ag 3d, (b) In 3d, (c) Ga 2p, (d) O 1s, (e) S 2p and (f) Al 2p of the AgIn_*x*_Ga_1−*x*_S_2_/GaS_*y*_ and AgIn_*x*_Ga_1−*x*_S_2_/GaS_*y*_ : Al synthesized with an Al/Ga molar ratio of 1 : 1.

The presence of Al was also confirmed by the XPS study. In Fig. 3, the Al 2p peak was observed at 74.1 eV in AgIn_*x*_G_1−*x*_S_2_/GaS_*y*_ : Al, corresponding to oxidized Al species such as Al–S or Al–O. However, these species are difficult to distinguish due to the low sensitivity of aluminum in XPS.^59^ The O 1s peak of AgIn_*x*_G_1−*x*_S_2_/GaS_*y*_ was located at 531.5 eV, corresponding to Ga–O, whereas the O 1s peak of AgIn_*x*_G_1−*x*_S_2_/GaS_*y*_ : Al QDs can be deconvoluted into two peaks with the binding energies of 531.8 eV and 530.1 eV, which are attributed to Ga–O and Al–O, respectively. These results suggest that Al atoms were doped into the GaS_*y*_ amorphous structure and they may have partially formed an oxide layer on the surface of the QDs.

**Scheme 1:**
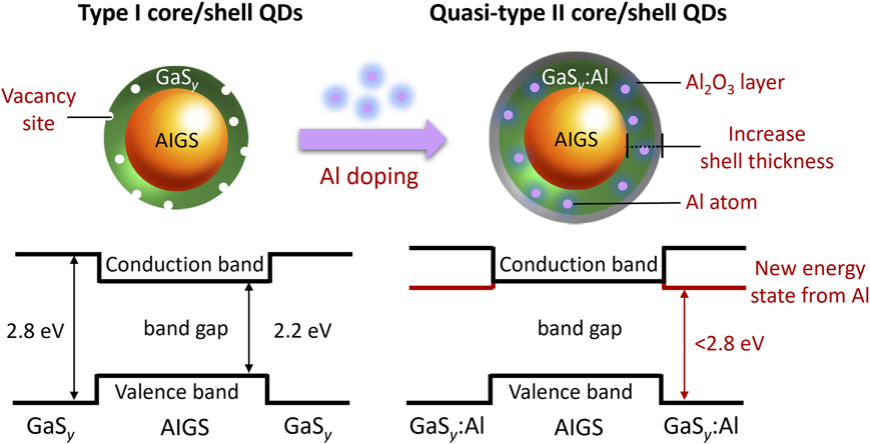
Schematic of the influence of Al on the morphology and energy band structure of AgIn_*x*_G_1−*x*_S_2_/GaS_*y*_ : Al core/shell QDs.

These structural characterizations demonstrate that Al ions can be successfully incorporated into the GaS_*y*_ shell without entering the AgIn_*x*_G_1−*x*_S_2_ core, as shown in Scheme 1. First, EDX analysis revealed that the Al content increased systematically with the precursor feed ratio, indicating effective control over Al incorporation during synthesis. This result is corroborated by X-ray photoelectron spectroscopy (XPS), which provides direct evidence for both the presence and chemical state of Al species. In particular, the appearance of a characteristic Al 2p signal, together with a deconvoluted component in the O 1s spectrum assigned to Al–O bonding, confirms that Al is present in an oxidized state within the material. Second, TEM images further demonstrated that increasing the Al/Ga ratio leads to a noticeable increase in both particle size and shell thickness. This observation is consistent with the expected growth behaviour, as Al^3+^ ions, possessing an ionic radius comparable to that of Ga^3+^, can be readily accommodated within the shell region, thereby promoting shell growth without significant lattice distortion. Third, XRD patterns showed no evidence of impurity phases or additional diffraction peaks across all samples. The absence of reflections corresponding to crystalline Al_2_O_3_, together with the lack of discernible peak shifts, suggests that Al neither forms a separate crystalline phase nor is substituted into the core lattice. These results collectively indicate that Al is predominantly incorporated into the amorphous shell, contributing to the observed structural evolution while preserving the integrity of the crystalline core.

**Fig. 4:**
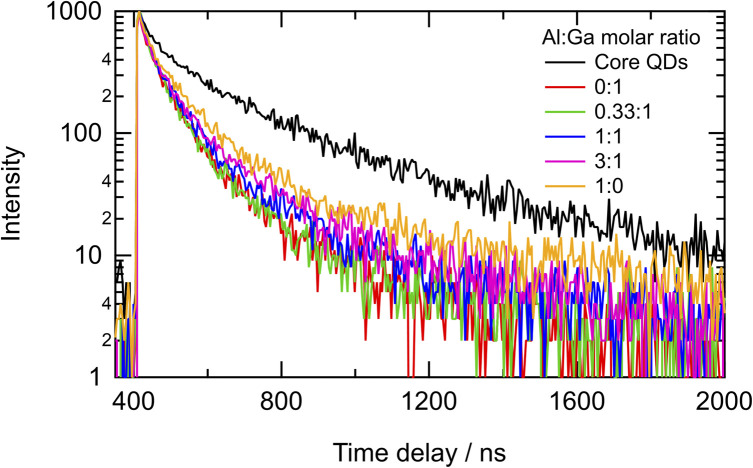
PL decay curves of AgIn_*x*_Ga_1−*x*_S_2_ core QDs and AgIn_*x*_Ga_1−*x*_S_2_/GaS_*y*_ : Al core/shell QDs synthesized using various Al/Ga molar ratios.

The PL QYs of core/shell QDs, which were measured using an integrating sphere, are summarized in Table 1. The PL QYs of core/shell QDs were improved by the doping of Al into the GaS_*y*_ shell. The best sample with the highest QY is that synthesized using a 1 : 1 Al/Ga molar ratio. However, when the Al/Ga molar ratio was increased, a decrease in PL QYs was observed due to the excess amount of Al, which would worsen the intrinsic chemical stability of the GaS_*y*_ : Al alloy and subsequently decrease the PL QYs of core/shell QDs. The enhancement of PL QY was deeply investigated using PL lifetime measurements. Fig. 4 shows the PL-decay curves of the AgIn_*x*_G_1−*x*_S_2_ core and AgIn_*x*_G_1−*x*_S_2_/GaS_*y*_ : Al core/shell QDs with various Al/Ga molar ratios. These curves could be well fitted using the following biexponential equation:8
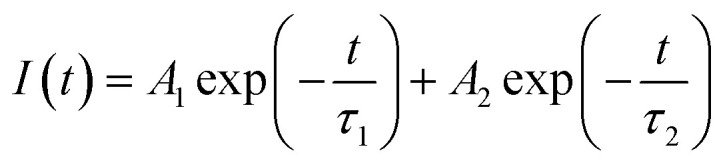
where *I*(*t*) is the intensity at time *t*; *A*_1_ and *A*_2_ represent the normalized amplitudes, and *τ*_1_ and *τ*_2_ represent the lifetime constants of the PL emission of each component.

A summary of the fitting results is presented in Table 3. For the AgIn_*x*_G_1−*x*_S_2_ core QDs, the fast (74 ns) and slow (352 ns) components are presented, which are shorter than the single lifetime component of AgInS_2_ QDs (*ca.* 1 µs) previously reported due to a greater nonradiative combination *via* the surface state in the alloy AgIn_*x*_G_1−*x*_S_2_ core.^32,47^ A decrease in lifetimes that originated from band-edge transitions occurred following the application of the GaS_*y*_ shell coating, with the values of 43 and 174 ns for the fast and slow components, respectively. The contribution of each fluorescence lifetime component was described in terms of the product of the amplitude and lifetime (*A* × *τ*). The fast component associated with band edge emission accounted for 58% of the total emission intensity, whereas the slow component associated with defect emission contributed to 42% of the total emission intensity. As the Al/Ga molar ratio increased, both lifetimes increased. However, the contribution of fast components gradually increased to 63% when the Al/Ga ratio reached 1 : 1. On the contrary, the contribution of slow components decreased to 37%. These findings suggest that non-radiative recombination centers might be suppressed by Al doping. The self-oxidation of these Al^3+^ ions can passivate the defect sites within the GaS_*y*_ shell, leading to an enhancement of PL QY.^44^ However, when the Al/Ga molar ratio increased to 3 : 1, the contribution of the fast component was reduced to 52%, while the slow component increased to 48%. This result confirms that an excess amount of Al may damage the intrinsic structure of the GaS_*y*_ shell, leading to a decrease in the PL QY. In addition, a lengthening of the lifetime can be observed when the Al ratio increases. This phenomenon might be related to two mechanisms. First, the non-radiative process can be modified by a thicker shell, which will cause the extension of the PL lifetime.^60^ On the other hand, when the shell gets thicker, the system will gradually evolve from type I to quasi-type II, and electrons will be located more in the shell. The spatial separation of electrons and holes will result in a decrease in the wave function overlap and thus, a longer radiative lifetime. Second, the Al^3+^ ions may modify the local electronic structure of the GaS_*y*_ shell by introducing a new energy state into the system.^61^ Therefore, the shell structure and thickness influence the exciton recombination process, as shown in Scheme 1.^62,63^

**Table 3:** PL decay components of AgIn_*x*_Ga_1−*x*_S_2_ core QDs and AgIn_*x*_Ga_1−*x*_S_2_/GaS_I_ : Al core/shell QDs synthesized using various Al/Ga molar ratios

Al/Ga molar ratio	PL decay						
	EMWL (nm)	CHI	〈*τ*〉 (ns)	*τ*_1_ (ns)	*τ*_2_ (ns)	*A*_1_	*A*_2_
Core NPs	596	1.20	292	74	352	452	339
0 : 1	507	0.93	98	43	174	873	156
0.33 : 1	512	0.84	109	49	202	797	123
1 : 1	520	1.0	111	49	216	826	110
3 : 1	530	0.97	146	52	251	758	141
1 : 0	535	1.11	190	69	354	764	109

### Effects of various shell precursor amounts for shell coating on the optical properties and morphology

The influence of the shell precursor quantity on the optical properties and morphologies of AgIn_*x*_G_1−*x*_S_2_/GaS_*y*_ was investigated using a 1 : 1 Al/Ga molar ratio for the shell coating. The DMTU and Al + Ga content ranged from 0.1 to 0.3 mmol. The UV-vis absorption and PL spectra of AgIn_*x*_G_1−*x*_S_2_/GaS_*y*_ : Al core/shell QDs are depicted in Fig. S1. With an increase in the quantity of precursors, the corresponding PL peak also shifted from 516 nm to 531 nm. The red-shift of the core/shell QDs can be attributed to electron wave function delocalization from the AgIn_*x*_G_1−*x*_S_2_ core into the GaS_*y*_ : Al shell, which is the characteristic behavior of quasi-type II core/shell QDs. Moreover, another significant change is the particle size, which increased with an increment of shell precursors, leading to a red-shift in optical spectra. The shell thickness increased from 2.4–3.4 nm when the shell precursor amount increased from 0.1 to 0.3 mmol, as shown in Fig. S2, which indicated the formation of a thicker shell on the AgIn_*x*_G_1−*x*_S_2_ core surface. Even though the quantity of precursors greatly increased, it exhibited a smaller effect on the particle size compared with the influence of the Al/Ga molar ratio. According to the EDX results (Table S1), the atomic ratios of Al were 0.68, 0.82, and 1.46, corresponding to 0.1, 0.2, and 0.3 mmol of shell amount, respectively, which is quite similar to the Al nominal atomic ratio (0.5, 1, and 1.5). However, the atomic ratio of Ga was lower than the nominal atomic ratio, which may be caused by the off-stoichiometry nature of the AgIn_*x*_G_1−*x*_S_2_ core synthesized from Ga(DDTC)_3_, as mentioned in the previous experiment.

Fig. S3 shows the powder X-ray diffraction patterns of AgIn_*x*_G_1−*x*_S_2_/GaS_*y*_ : Al core/shell QDs synthesized using various shell precursor amounts. All the samples exhibited three similar peaks corresponding to the AgIn_*x*_G_1−*x*_S_2_ core QDs structure. Even though the quantity of shell precursors was tremendously increased, the planes of GaS_*y*_ or Al_2_O_3_ were still not observed in the XRD patterns. It was confirmed that increasing the shell precursor amounts does not destroy the intrinsic properties of the AgIn_*x*_G_1−*x*_S_2_ core and GaS_*y*_ shell. This phenomenon may be attributed to an increase in PL QYs while the precursor amounts were increased, as shown in Table S2. The overall PL QYs increased to 41%, and the value was 29.6% when the wavelength region was limited to the band-edge emission for the 0.3 mmol conditions. The PL QY of this sample is approximately 3 times greater than that of the AgInS_2_/GaS_*y*_ core/shell QDs encapsulated in indium-fumarate MOFs.^38^ Based on this surface modification method, we can double the PLQYs of the AgIn_*x*_G_1−*x*_S_2_/GaS_*y*_ core/shell QDs and still maintain their emission, which is very useful for practical applications.

The PL decay curves corresponding to this study are shown in Fig. S4. It was found that for all samples, their PL decay curves could be well fitted with an exponential equation, as in a previous study. The fast component was derived from band-edge emission due to the GaS_*y*_ : Al shell coating, whereas the slow component was derived from defect emission. On increasing the shell precursor amount, the PL lifetimes were lengthened, as shown in Table S3.

### Effects of Al/Ga molar ratios and different shell precursor amounts for shell coating on stability

**Fig. 5:**
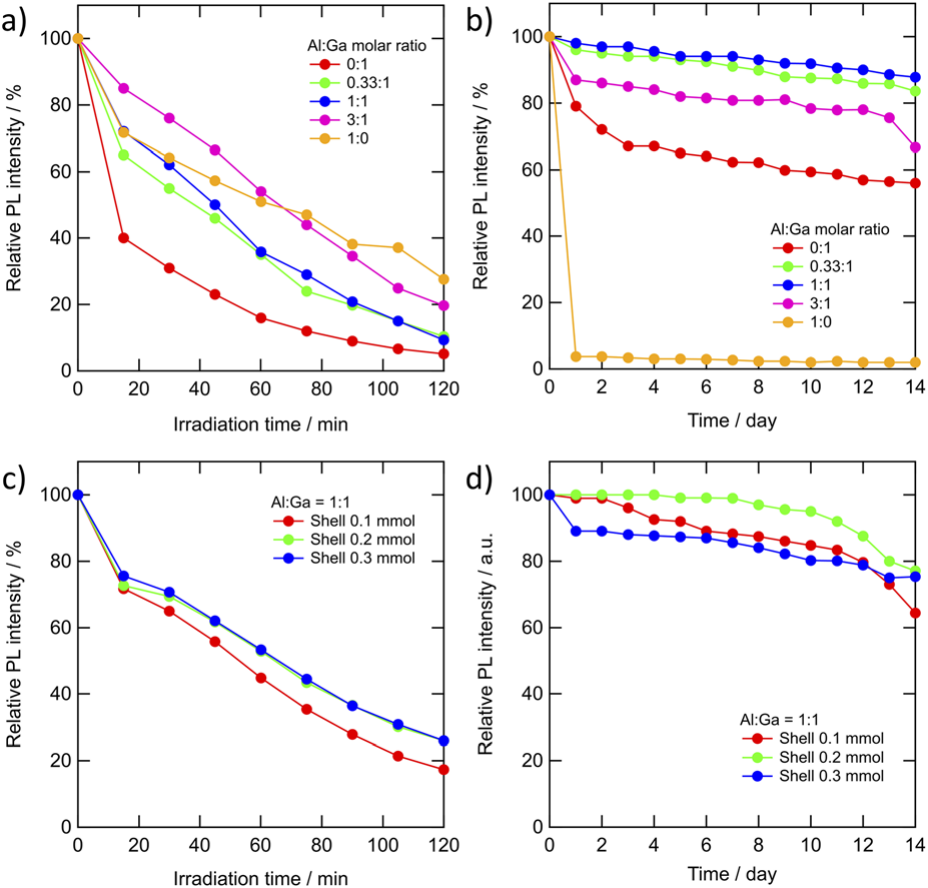
(a and c) Photostability and (b and d) long-term stability for (a and b) AgIn_*x*_Ga_1−*x*_S_2_/GaS_*y*_ : Al QDs synthesized using various Al/Ga molar ratios and (c and d) AgIn_*x*_Ga_1−*x*_S_2_/GaS_*y*_ : Al QDs synthesized using various shell precursor amounts.

Photostability and long-term storage stability tests were carried out for AgIn_*x*_G_1−*x*_S_2_/GaS_*y*_ and AgIn_*x*_G_1−*x*_S_2_/GaS_*y*_ : Al core/shell QDs, as shown in Fig. 5. After being irradiated by the UV lamp (360 nm, 1 mW cm^−2^) for 120 min, the AgIn_*x*_G_1−*x*_S_2_/GaS_*y*_ : Al samples degraded slower than AgIn_*x*_G_1−*x*_S_2_/GaS_*y*_ QDs, indicating that Al doping can protect the core/shell QDs from photooxidation induced by UV light (Fig. 5a). In addition, the sample synthesized using a 1 : 1 ratio maintained its PL intensity at about 80% for 14 days, which is longer than the AgInS_2_/GaS_*y*_ core/shell QDs encapsulated by indium-fumarate MOFs (7 days).^38^ The sample without Al maintained only 60% (Fig. 5b). These results confirm the influence of Al doping in the GaS_*y*_ shell toward the stability of these core/shell particles. With increased shell precursor amounts, after exposure to UV light, the photostability was found to increase (Fig. 5c); moreover, it increased the long-term storage stability, as shown in Fig. 5d. Because increasing the amount of shell precursor leads to increased shell thickness, it is possible that the thicker shell and the Al_2_O_3_ formation can improve the photostability and durability of these core/shell QDs.

### Methyl orange adsorption study of the QDs@P25–TiO_2_ composite

**Fig. 6:**
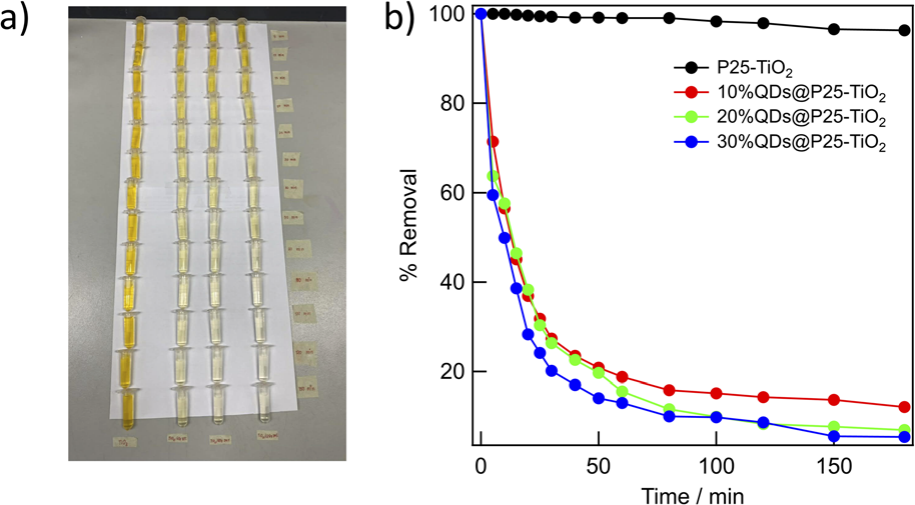
(a) An image of the aliquots, and (b) the removal rates of P25–TiO_2_ and QDs@P25–TiO_2_ composites during the adsorption process of methyl orange.

The adsorption property of the composite material was determined using methyl orange as a test dye. Several parameters affecting the removal of methyl orange were studied to investigate the adsorption mechanism between QDs@P25–TiO_2_ and methyl orange, including the QDs to P25–TiO_2_ ratio in the composite, contact time, and initial dye concentration. Dimethylaminoazobenzenesulfonate or methyl orange is an azobenzene derivative that presents a bright orange color when dissolved in water. Typically, the absorption spectrum of methyl orange displays two absorption peaks, with the main peak at approximately 510 nm and a smaller peak around 465 nm. The absorption is affected by pH; as the pH increases, the peak at 510 nm decreases while the peak at 465 nm increases, causing a color change from red (acidic form) to yellow (basic form).^64,65^ When the P25–TiO_2_ was added to the methyl orange solution, the color of the dye remained unchanged, which means there was no change in pH after the addition, as shown in Fig. 6a. According to Fig. S5a, in the absorption spectra of methyl orange after the addition of P25–TiO_2_, only a slight decrease in absorbance at 465 nm was observed without any shift in the position of the peak during the adsorption process. The results indicate that P25–TiO_2_ by itself cannot absorb or react with methyl orange. This phenomenon would limit the synergistic effect of absorption-photodegradation, which is necessary for photocatalysis in wastewater treatment. On the contrary, when the QDs@P25–TiO_2_ composite was added to the dye solution, the orange colour of the methyl orange solution gradually faded during the reaction process, as shown in Fig. 6a. In addition, the absorbance of the peak relating to basic methyl orange decreased with time, indicating that the methyl orange was adsorbed by the composite materials, as shown in Fig. S5b–d. The effect of the QD content in the composite on the removal rate of methyl orange is shown in Fig. 6b. Initially, all samples rapidly adsorbed methyl orange by more than 50% in 15 min. The 30%QDs@P25–TiO_2_ composite exhibited the fastest removal rate compared to the others in the first phase of reaction. This may be attributed to the increased number of active sites arising from the higher QD content in the composite. The concentration of the remaining methyl orange gradually decreased and then became constant at 80 min under all conditions. Upon completion of the adsorption process (180 min), the removal rates were 11.9%, 6.8%, and 5.3% for 10%, 20%, and 30% QDs@P25–TiO_2_ composites, respectively, indicating negligible efficacy. Therefore, a minimal concentration of quantum dots on TiO_2_ significantly improves the adsorption capacity of the composite for wastewater treatment applications.

The adsorption isotherm and kinetic studies were performed using the 10%QDs@P25–TiO_2_ composite as an adsorbent. The initial concentrations of methyl orange varied from 5 to 40 mg L^−1^. The data for the adsorption of methyl orange by the 10%QDs/P25–TiO_2_ composite were fitted according to the Langmuir and the Freundlich adsorption isotherm models. The isotherms were plotted as shown in Fig. S6. Typically, the Langmuir adsorption isotherm model describes monolayer adsorption in a homogeneous surface, while the Freundlich model describes multilayer adsorption in a heterogeneous surface.^66^ The correlation coefficient (*R*^2^) in the Langmuir isothermal adsorption model for the adsorption process was 0.89, whereas *R*^2^ for the Freundlich adsorption isotherm model was 1.00. The higher *R*^2^ of the Freundlich adsorption model suggests that the adsorption of methyl orange by the 10%QDs/P25–TiO_2_ composite was more consistent with this model, primarily exhibiting multilayer adsorption characteristics. Additionally, for methyl orange dye, the 1/*n* value was 0.49, indicating a favorable adsorption process. Therefore, the QDs@P25–TiO_2_ composite may be a useful adsorbent for the removal of methyl orange. Typically, the adsorption mechanism consists of three stages. Initially, the methyl orange molecules diffuse to interact with the surface of the composite material. Subsequently, the methyl orange molecules permeate into the pores of the QDs@P25–TiO_2_. Finally, methyl orange molecules are bound at specific adsorption sites of the adsorbent to complete the adsorption process.^67^ For further investigation of the mechanism in methyl orange removal, the experimental data were fitted to the pseudo-first-order and pseudo-second-order kinetic models using eqn (6) and (7). The results are presented in Fig. S7 and Table S4. The pseudo-first-order *R*^2^ values for the adsorption of methyl orange onto the composite were 0.95, 0.99, 0.95, 0.88, and 0.98 for 5, 10, 20, 30, and 40 mg L^−1^ of methyl orange, respectively. Whereas, the pseudo-second-order kinetic model coefficients *R*^2^ were 1.00 for all composite materials. In addition, the calculated equilibrium adsorption capacities from the pseudo-second-order kinetic models for methyl orange were close to the experimental values. The data indicate that the adsorption process of methyl orange onto QDs@P25–TiO_2_ follows the pseudo-second-order kinetic model more closely, which suggests that the interaction between the material and the dyes is the rate-controlling step. The results show that this is a chemisorption process, which may be attributed to the interaction between OLA on the surface of QDs and methyl orange.^68^

## Conclusions

In summary, the PL properties and stability of AgIn_*x*_G_1−*x*_S_2_/GaS_*y*_ core/shell QDs were successfully enhanced *via* a simple Al-doping approach. The incorporation of Al into the GaS_*y*_ shell increased the shell thickness, while subsequent oxidation led to the formation of a protective Al_2_O_3_ layer. This layer effectively shielded the QDs from environmental degradation, resulting in improved PL QY and long-term durability. Moreover, the Al_2_O_3_ may diminish the defect states of GaS_*y*_, resulting in the suppression of non-recombination processes, thus enhancing the emission of the nanoparticles. A red shift in the emission was observed as a result of increased particle size and the introduction of additional states, caused by Al doping, which facilitated a transition from type-I to quasi-type-II core/shell band alignment. Owing to these advantages, AgIn_*x*_G_1−*x*_S_2_/GaS_*y*_ : Al core/shell QDs are promising candidates for bandgap engineering of TiO_2_. Focusing on the adsorption behavior of the QDs/P25–TiO_2_ composite, the anionic methyl orange molecule was used as a model organic pollutant. The composite exhibited a significantly higher removal efficiency compared with bare P25–TiO_2_. Adsorption isotherm analysis revealed good agreement with the Freundlich model, indicating a heterogeneous surface resulting from the presence of QDs on the P25–TiO_2_ surface. Additionally, kinetic analysis demonstrated that the adsorption process followed a pseudo-second-order model, suggesting a chemisorption mechanism between methyl orange molecules and the QDs/P25–TiO_2_ composite. These findings suggest that AgIn_*x*_G_1−*x*_S_2_/GaS_*y*_ : Al core/shell QDs have significant potential for TiO_2_ functionalization as dye adsorbents, thereby laying a solid foundation for the future development of wastewater treatment.

## Author contributions

M. T.: investigation, formal analysis, data curation, visualization, conceptualization, writing–original draft. S. N.: investigation, formal analysis, data curation. V. T.: investigation, data curation. P. K.: investigation, data curation. N. G.: investigation, data curation. A. R.: investigation, data curation. W. M.: conceptualization, resources, writing–review and editing, supervision, funding acquisition.

## Conflicts of interest

There are no conflicts to declare.

## Supplementary Material

RA-OLF-D6RA00895J-s001

## Data Availability

All data supporting the findings of this study are available within the article and its supplementary information (SI). Supplementary information is available. See DOI: https://doi.org/10.1039/d6ra00895j.
