# Supplementary material for: Aluminum-doped gallium sulfide shell for enhancing the luminescence properties of Ag-In-Ga-S core quantum dots and their composite for dye adsorption
Source: RSC Adv. 2026 Jul 2;16(34):32018–28. doi: 10.1039/d6ra00895j (PMC13325000; doi:10.1039/d6ra00895j)
Supplement: RA-016-D6RA00895J-s001 [file RA-016-D6RA00895J-s001.pdf]

Supplementary Information (SI) for RSC Advances.
This journal is © The Royal Society of Chemistry 2026

Supplementary Information

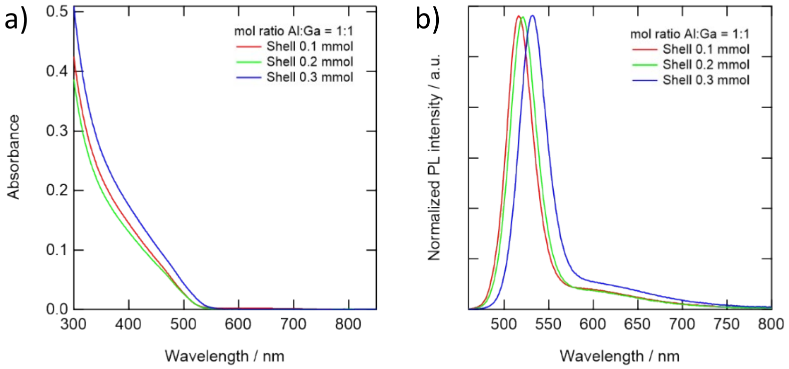

Figure S1 (a) UV-vis absorption and (b) intensity-normalized PL spectra for $AgIn_xGa_{1-x}S_2$, and $AgIn_xGa_{1-x}S_2/GaS_y$:Al QDs synthesized using various amounts of shell precursors.

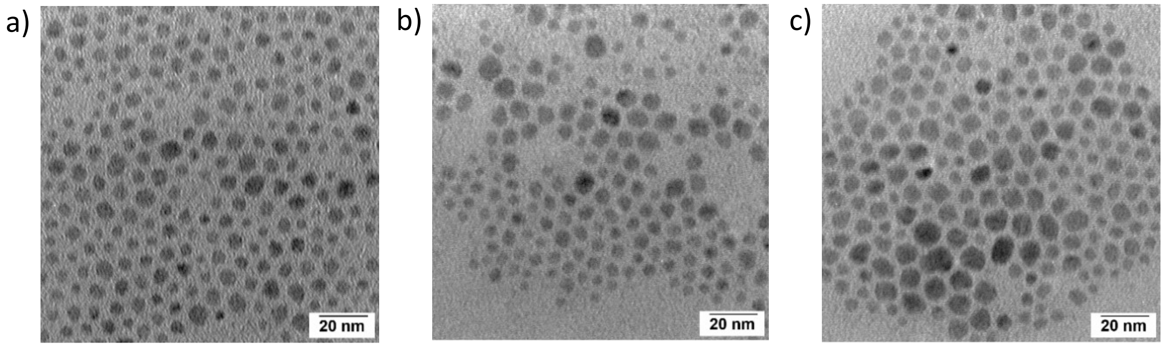

Figure S2 TEM images of $AgIn_xGa_{1-x}S_2/GaS_y$:Al QDs with (a) 0.1 mmol, (b) 0.2 mmol and (c) 0.3 mmol of total amount of shell precursors.

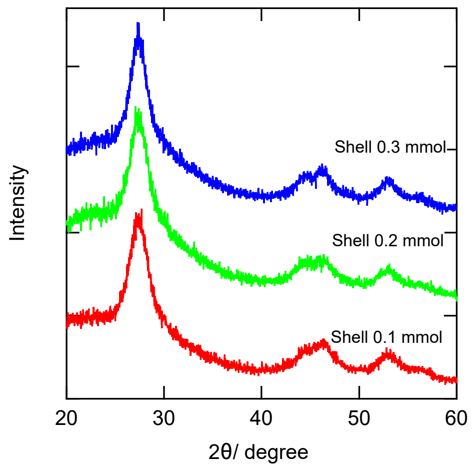

Figure S3 XRD patterns for $AgIn_xGa_{1-x}S_2/GaS_y$:Al core/shell QDs synthesized using various shell precursor amounts.

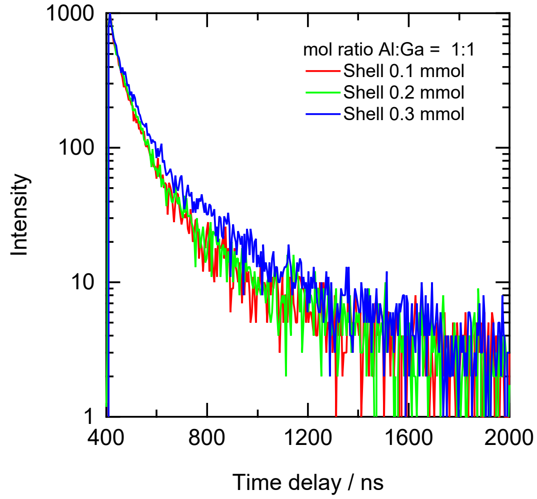

Figure S4 PL decay curves of $AgIn_xGa_{1-x}S_2/GaS_y$:Al core/shell QDs synthesized using various shell precursor amounts.

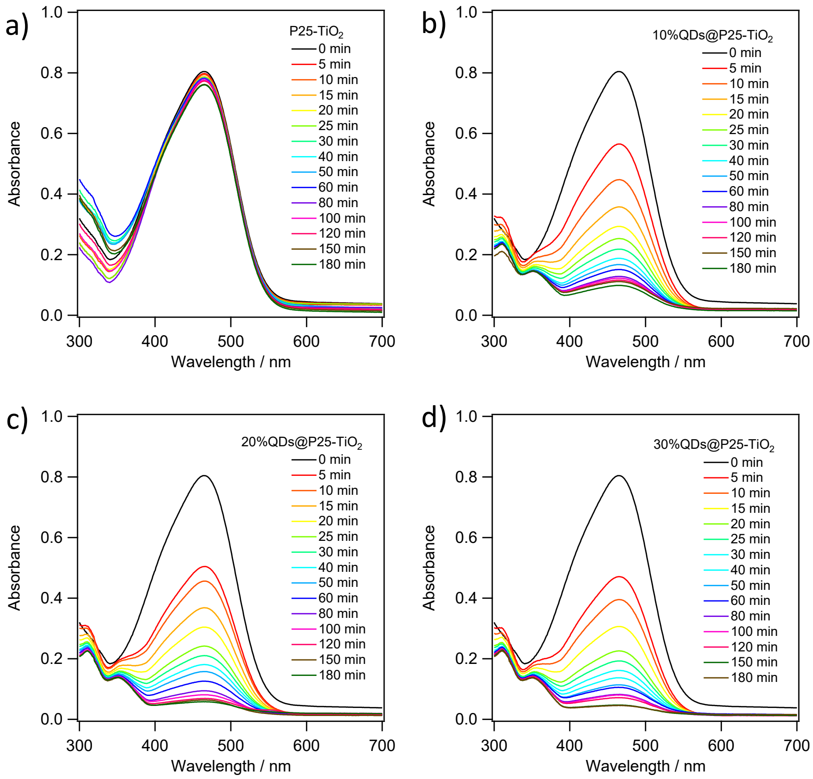

Figure S5 Absorption spectra of 10 mg/L methyl orange treated with (a) P25-TiO_2, (b) 10%QDs@P25-TiO_2, (c) 20%QDs@P25-TiO_2, and (d) 30%QDs@P25-TiO_2 at different time intervals.

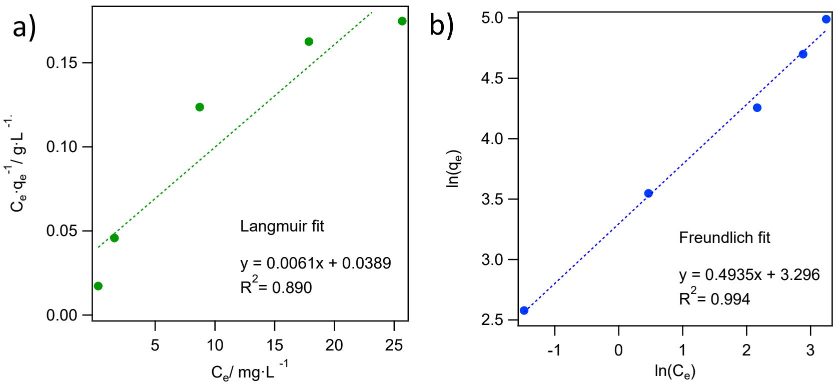

Figure S6 Linear fitting graphs of Langmuir (a) and Freundlich (b) equations for 10%QDs@P25-TiO_2 composite adsorption of methyl orange.

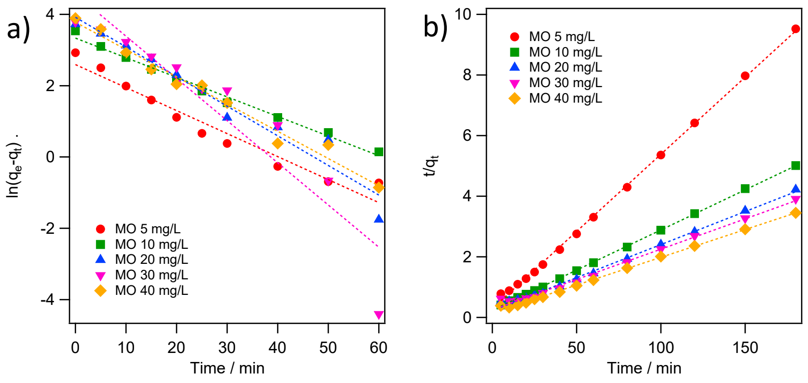

Figure S7 The adsorption kinetics of methyl orange toward 10%QDs@P25-TiO_2 composite with different initial concentration of dye.

Table S1 Atomic concentration of $AgIn_xGa_{1-x}S_2/GaS_y$:Al core/shell QDs synthesized using various shell precursor amounts

Shell amount (mmol)	Al	S	Ag	In	Ga
0.1	0.68	2.63	1.00	0.43	0.86
0.2	0.82	2.69	1.00	0.41	0.79
0.3	1.46	2.94	1.00	0.48	1.04

Table S2 Relevant parameters of $AgIn_xGa_{1-x}S_2/GaS_y$:Al core/shell QDs synthesized using various shell precursor amounts

Shell amount (mmol)	Peak location (nm)	PL QY (%)	Particle size (nm)	Shell thickness (nm)
**0.1**	517	22.4 31.0	6.0±1.3	2.4
**0.2**	521	26.7 35.2	6.6±1.6	3.0
**0.3**	532	29.6 41.0	7.0±1.7	3.4

Table S3 PL decay components of $AgIn_xGa_{1-x}S_2/GaS_y$:Al core/shell QDs synthesized using various shell precursor amounts

Shell amount	PL decay						
	EMWL (nm)	CHI	<τ> (ns)	τ_1 (ns)	τ_2 (ns)	A_1	A_2
0.1 mmol	516	1.0	111	49	216	826	110
0.2 mmol	521	0.9	117	52	237	842	101
0.3 mmol	531	0.9	151	57	271	780	127

Table S4 Pseudo-first order and pseudo-second-order kinetic parameters of 10%QDs@P25-TiO_2 composite toward methyl orange

Initial concentration of MO	Pseudo-first order fitting			Pseudo-second order fitting			Experime-ntal q_e (mg/g)
	k_1 (1/min)	q_e (mg/g)	R^2	k_2 (g/mg min)	q_e (mg/g)	R^2	
5 mg/L	0.0645	13.38	0.95	0.0088	19.65	1.00	18.63
10 mg/L	0.0549	28.02	0.99	0.0029	37.74	1.00	34.42
20 mg/L	0.0831	50.28	0.95	0.0021	45.87	1.00	41.43
30 mg/L	0.1184	96.77	0.88	0.0016	50	1.00	43.91
40 mg/L	0.0746	43.68	0.98	0.0022	54.64	1.00	49.04
